# Psychometrics and diagnostics of the Italian version of the Beck Depression Inventory-II (BDI-II) in Parkinson’s disease

**DOI:** 10.1007/s10072-023-06619-w

**Published:** 2023-01-18

**Authors:** Gianpaolo Maggi, Alfonsina D’Iorio, Edoardo Nicolò Aiello, Barbara Poletti, Nicola Ticozzi, Vincenzo Silani, Marianna Amboni, Carmine Vitale, Gabriella Santangelo

**Affiliations:** 1 Department of Psychology, University of Campania “Luigi Vanvitelli”, Caserta, Italy; 2 Department of Neurology and Laboratory of Neuroscience, IRCCS, Istituto Auxologico Italiano, Milano, Italy; 3 PhD Program in Neuroscience, School of Medicine and Surgery, University of Milano-Bicocca, Milan, Italy; 4 Department of Pathophysiology and Transplantation, “Dino Ferrari Center”, Università degli Studi di Milano, Milano, Italy; 5 Institute of Diagnosis and Health, IDC-Hermitage Capodimonte, Naples, Italy; 6 Department of Medicine, Surgery and Dentistry, University of Salerno, Salerno, Italy; 7 Department of Motor Sciences and Wellness, University “Parthenope”, Naples, Italy

**Keywords:** Beck Depression Inventory, Parkinson’s disease, Depression, Anxiety, Diagnostics, Psychometrics

## Abstract

**Introduction:**

Depression is one of the most disabling neuropsychiatric manifestations of Parkinson’s disease (PD) and requires proper screening and diagnosis because it affects the overall prognosis and quality of life of patients. This study aimed to assess the psychometric and diagnostic properties of the Beck Depression Inventory-II (BDI-II) in an Italian PD cohort.

**Materials and methods:**

Fifty consecutive outpatients with PD underwent the Italian version of the BDI-II and other questionnaires to evaluate anxiety and apathetic symptoms. Patients’ caregivers completed the depression/dysphoria domain of the Neuropsychiatric Inventory (NPI-D). We evaluated the internal consistency, convergent and divergent validity, and factorial structure of BDI-II. Sensitivity, specificity, positive and negative predictive values, and likelihood ratios were computed using ROC analyses, and an optimal cutoff was defined using the Youden index.

**Results:**

The BDI-II proved to be internally consistent (Cronbach’s *α* = 0.840) and substantially met the bi-factorial structure. Regarding construct validity, the BDI-II was substantially related to anxiety measures, but not to apathy. With the combination of the NPI-D and anxiety score used as the gold standard, the BDI-II overall showed good accuracy (AUC = 0.859) with adequate sensitivity (75%) and specificity (87%). The optimal cutoff point was defined at 14.50.

**Conclusions:**

We provide evidence of the psychometric and diagnostic properties of the Italian version of the BDI-II as a screening tool for depression in patients with PD. The BDI-II was found to be reliable and valid for the measurement of depression in patients with PD; therefore, it is available for use in clinical research and practice.

**Supplementary Information:**

The online version contains supplementary material available at 10.1007/s10072-023-06619-w.

## Introduction

Depression represents one of the most frequent and disabling non-motor manifestations of Parkinson’s disease (PD) because its prevalence ranges from 17 to 38% [1, 2] and it heavily affects patients’ quality of life [3]. More specifically, the risk of depression in PD is more than double that in the general population (15–18%) [4], suggesting that this manifestation could also be attributable to disease-related factors.

Even though a unified pathophysiological model of depression in PD, considering the interaction with incidental depression after diagnosis, is still lacking [5], its onset may precede the motor manifestations [6] and affects patients’ overall prognosis [7].

A distinct profile of depressive symptoms was observed in PD patients compared to non-PD cohorts [8–10]. Particularly, depression in PD is characterized by fewer feelings of guilt, attempted suicide, and less severe sadness but more severe somatic symptoms such as fatigue and concentration difficulties [8, 9]. Within this context, early detection of depressive symptomatology results is clinically crucial to timely plan targeted pharmacological [11] and/or non-pharmacological interventions [12].

However, its psychometric assessment and diagnosis in this population are challenged by the confounding effects of both motor disabilities and behavioral mimics (e.g., apathy) [13]. Another confounder is represented by the frequent co-existence of depression and anxiety [14, 15], which moderately overlap as to their clinical manifestations and share common pathophysiological underpinnings [16]. Therefore, it is not advisable that common instruments assessing depression be straightforwardly applied to PD patients; in contrast, disease-specific psychometrics and diagnostics of such instruments should be derived to be validly applied in both clinical practice and research.

Out of these, the Beck Depression Inventory-II (BDI-II) has demonstrated strong psychometric properties and may be useful for depression screening in the PD population [17]. Although the BDI-II includes somatic items that may increase the BDI-II score in PD population, these do not seem to decrease the ability to discriminate between depressed and non-depressed patients [18].

Given the above premises, this study aimed at providing psychometric and diagnostic properties for the BDI-II in an Italian PD cohort

## Materials and methods

### Participants

Fifty consecutive PD outpatients were recruited at Movement Disorders Unit of IDC-Hermitage Capodimonte in Naples, according to the following inclusion criteria: (i) diagnosis of idiopathic PD based on the clinical diagnostic criteria [19]; (ii) absence of other neurological and psychiatric conditions; and (iii) absence of cognitive decline [20].

We collected demographic (i.e., age, sex, years of schooling) and clinical data as the years from diagnosis, the severity of motor symptoms assessed by part III of the Unified Parkinson’s Disease Rating Scale (UPDRS-III), the stage of the disease using the Hoehn & Yahr staging system (H&Y), and the levodopa equivalent daily dose (LEDD).

This study was approved by the Local Ethics Committee and conducted following the ethical standards of the Declaration of Helsinki and its later amendments. Participants provided informed consent and data were treated according to current regulations.

### Materials

Patients completed the Italian version of the BDI-II [21], the Parkinson Anxiety Scale (PAS) [22], the Apathy Evaluation Scale (AES) [23], and the Montreal Cognitive Assessment (MoCA) [20]. Moreover, patients’ caregivers completed the depression/dysphoria domain of the Neuropsychiatric Inventory (NPI-D) [24].

The BDI-II is a self-report questionnaire developed to measure depressive symptomatology [25] and consists of 21 items presented in the form of statements organized according to the severity of their content. Respondents are asked to select the most appropriate statement to describe their mood in the last 2 weeks. These are measured on a 4-point scale from a minimum of 0 points to a maximum of 3, with higher scores indicating greater severity of depressive symptoms. Its administration takes 5/10 min.

The PAS is a 12-item observer or patient-rated scale developed to evaluate anxiety symptoms in PD patients [26]. It consists of three subscales that measure persistent anxiety, episodic anxiety, and avoidance behavior. Respondents are asked to indicate the severity/frequency of their anxiety symptoms during the past 4 weeks from 0 (not at all/never) to 4 (severe/almost always) with higher scores indicating more severe anxiety.

The AES is a self-report 18-items scale assessing behavioral, cognitive, emotional, and other aspects of apathy [23]. Responses are measured on a 4-point Likert-type scale (“Not at all,” “Slightly,” “Somewhat,” and “A lot”) with reverse scoring for some items. Items worded with positive syntax are recorded and the total score ranges from 18 to 72 points, with higher scores indicating more severe apathy.

The NPI-D is a relatively brief interview with the patient’s caregiver or someone who knows the patient well and begins with a yes/no screening question about the presence of depressive symptoms. If the screen is positive, eight subquestions are then administered and the caregiver scores the frequency and severity of these symptoms. The final scoring is based on the frequency (1–4) × severity (1–3) product.

### Statistics

According to previous investigations on the standardization of behavioral scales [27], acceptability of the BDI-II was defined as appropriate in the absence of missing values, and floor/ceiling effects were ruled out if the scores did not exceed a skewness value of |2|.

Construct validity was investigated via Bonferroni-corrected Spearman’s correlations due to the non-normality of BDI-II scores—i.e., skewness and kurtosis values ≥ |1| and |3|, respectively [28]. Non-parametric techniques were adopted to test the association between BDI-II scores and demographic/clinical variables.

Internal consistency was tested using Cronbach’s *α*. The bi-factorial structure assumed to underpin the BDI-II [29] was explored via an OBLIMIN-rotated, regularized exploratory factorial analysis (REFA), which is a dimensionality-reduction technique designed for small sample sizes (*N* ≤ 50) [30]. A regularized least squares approach was adopted to estimate loadings.

Diagnostics were assessed via receiver-operating characteristic (ROC) analyses, by computing both intrinsic—sensitivity (Se) and specificity (Sp)—and post-test properties—positive and negative predictive values (PPV; NPV) and likelihood ratios (LR+; LR−)—at the optimal cutoff identified using Youden’s *J* statistic. To achieve this, by conservatively forecasting a prevalence of clinically meaningful depression of 20% [1], the minimum sample sizes were estimated, according to Goksuluk et al. [31], at *N* = 6 and *N* = 25 for depressed and non-depressed patients, respectively (allocation ratio: 4), by addressing the following parameters: *α* =.05, 1-β = .8, AUC = .8. Within ROC analyses, the positive outcome was operationalized as a combination of (i) an NPI-D score above the 75th percentile of the empirical distribution and (ii) a PAS score above the established cutoff [22]. Such a choice to derive the reference measure from both depression and anxiety indexes has been made based on the fact that (1) the two disorders overlap to a moderate extent as to their clinical manifestations and (2) are known to frequently co-occur and share common pathophysiological mechanisms in PD patients [14, 15].

## Results

Table 1 shows the demographic and clinical characteristics of the patients. Acceptability rate was 100%, and no marked floor effects were detected (skewness = 1.235). At *α*_adjusted_ = 0.025, BDI-II scores were unrelated to age (*r*_*s*_ = 0.053; *p* = 0.714), education (*r*_*s*_ = − 0.224; *p* = 0.119), and sex (*U* = 259.00; *p* = 0.941). As for clinical variables, the BDI-II scores were not associated with disease duration (*r*_*s*_ = 0.232; *p* = 0.161), H&Y stage (*r*_*s*_ = 0.274; *p* = 0.143), UPDRS-III (*r*_*s*_ = 0.311; *p* = 0.058), and LEDD (*r*_*s*_ = 0.242; *p* = 0.149) at *α*_adjusted_ = 0.0125.

**Table 1:** Descriptive statistics on demographic, clinical, and neuropsychological variables

	Mean ± SD
Age (*ys*)	65.16 ± 8.65
Education (*ys*)	11.22 ± 4.52
Gender (*n*)	*M* = 35; *F* = 15
Disease duration (*ys*)	11.00 ± 5.68
UPDRS-III	14.74 ± 7.91
Hoehn & Yahr	2.45 ± 0.66
LEDD	781.76 ± 371.91
MoCA	19.60 ± 4.46
BDI-II	8.44 ± 6.70
NPI-D	2.34 ± 3.34
PAS	10.26 ± 9.04
AES	34.29 ± 10.61

*SD*, standard deviation; *ys*, years; *n*, number; *UPDRS*, Unified Parkinson’s Disease Rating Scale; *LEDD*, Levodopa Equivalent Daily Dose; *MoCA*, Montreal Cognitive Assessment; *BDI-II*, Beck Depression Inventory – II; *NPI-D*, Depression/dysphoria domain of the Neuropsychiatric Inventory; *PAS*, Parkinson Anxiety Scale; *AES*, Apathy Evaluation Scale

At *α*_adjusted_ = 0.0125, the BDI-II scores were positively related to both PAS (*r*_*s*_ = 0.675; *p* < .001) and NPI-D scores (*r*_*s*_ = 0.401; *p* = 0.004), but not with AES (*r*_*s*_ = 0.265; *p* = .079), and MoCA (*r*_*s*_ = − 0.113; *p* = 0.435) scores.

The BDI-II proved to be internally consistent (Cronbach’s *α* = 0.840) and substantially met a bi-factorial, oblique (correlation between factors: *r* = .3) structure according to the REFA (Supplementary Material 1)—with 58.52% of variance explained and loadings on both factors ≥ .3

**Fig. 1:**
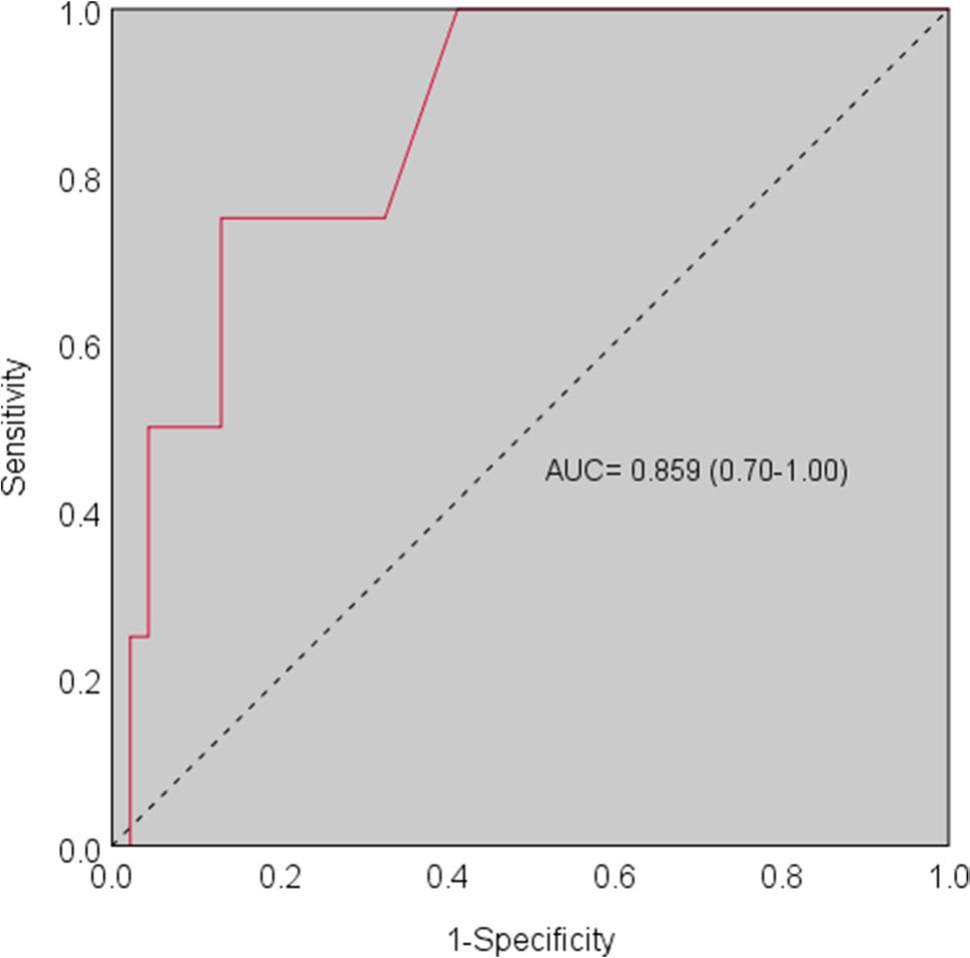
ROC curve for the BDI-II

Four patients (8%) were classified as positive according to the present operationalization of depression. The BDI-II yielded, at an optimal cutoff of 14.5 (*J* = 0.620), high accuracy in discriminating depressed from non-depressed PD patients (AUC = 0.859; *SE* = 0.078; CI 95% [0.705, 0.999]), with adequate both intrinsic (Se = 0.750; Sp = 0.870) and post-test properties (PPV = 0.333; NPV = 0.976; LR+ = 5.750; LR− = 0.288) (Fig. 1). Based on the abovementioned cutoff (< 15: not depressed; ≥ 15: depressed), 9 patients were classified as depressed (18%).

## Discussion

The present study provides the first evidence of the psychometric and diagnostic soundness of the Italian version of the BDI-II as a screening tool for depression in PD patients.

The BDI-II demonstrated both convergent and divergent validity, as proved by significant relationships with depression (NPI-D) and anxiety (PAS) measures but not with apathy (DAS) and global cognitive (MoCA) ones. Taken together, these findings further support the idea that apathy represents a distinguishable clinical phenomenon that occurs also in the absence of depression [32–35]. Moreover, our choice to derive the reference measure for ROC analysis using a combination of data from NPI-D and PAS is further corroborated by the strong association between depression and anxiety, which frequently co-occur in PD due to the overlap of clinical manifestations and pathophysiological mechanisms [14, 15].

Conversely, the absence of a relationship between depression and poorer cognition contrasts with previous studies that revealed more severe global cognitive dysfunction in depressed PD patients [1]. Nevertheless, it should be noted that most of our participants experienced mild to moderate depressive symptoms, which may act as a confounding factor since cognitive dysfunctions are usually linked to more severe depressive manifestations.

As for clinical variables, the BDI-II scores were not associated with PD duration or severity. Indeed, depressive symptoms can also occur in the early stage of the disease [36] and without differences in their prevalence between clinical phenotypes [37, 38], suggesting that their presentation is sustained not only by specific disease-related factors. Particularly, it has been observed that the effect of PD-specific factors on depression is smaller than that of a number of general risk factors unrelated to PD (i.e., preexisting vulnerabilities, premorbid personality, and life events) [39]; thus, future studies on the etiology of depression in PD should adopt a broad multifactorial approach to investigate the contribution of nonspecific markers.

Moreover, the BDI-II proved to be internally consistent and met an expected bi-factorial structure. Our results substantially confirm the bi-factorial structure reflecting Somatic-affective and Cognitive symptoms, frequently obtained using clinical samples [40], despite some differences in the partition of items into factors, likely due to the limited number of patients included. However, it should be noted that the factorial structure of the BDI-II still remains controversial and inconsistent across the studies [41] positing the existence of alternative structural models [42].

The BDI-II also provided optimal intrinsic and post-test diagnostic properties, with the latter playing a relevant role in orienting clinicians’ decisions such as excluding the diagnosis of depression and carrying out further examination considering its prevalence in PD (PPV; NPV) and independently of the clinical population (LR+; LR−). A cutoff of ≥ 15 is proposed to detect depression.

This study is not fully exhaustive regarding the clinicometric properties of BDI-II in patients with PD. Future investigations are needed to confirm these data by comparing the PD population to a healthy control group. In addition, test-retest and inter-rater reliability are still to be tested. Longitudinal studies are advised to provide reliable measures and evidence of sensitivity to change. A further limitation to be accounted for is represented by the fact that a specific operationalization of depression has been addressed. Future investigations aimed at confirming the present findings by adopting different reference measures are advisable. In conclusion, we demonstrated that the BDI-II is a valid, reliable, and diagnostically sound screener for depressive symptomatology in PD patients, and its adoption is encouraged in clinical practice and research.

## Supplementary information

ESM 1 (DOCX 16 kb)

## Data Availability

Datasets associated with the present study are available upon reasonable request of interested researchers.
