# Correction to: Psychometrics and diagnostics of the Italian version of the Beck Depression Inventory-II (BDI-II) in Parkinson’s disease

**DOI:** 10.1007/s10072-023-06746-4

**Published:** 2023-03-20

**Authors:** Gianpaolo Maggi, Alfonsina D’Iorio, Edoardo Nicolò Aiello, Barbara Poletti, Nicola Ticozzi, Vincenzo Silani, Marianna Amboni, Carmine Vitale, Gabriella Santangelo

**Affiliations:** 1 Department of Psychology, University of Campania “Luigi Vanvitelli”, Caserta, Italy; 2 Department of Neurology and Laboratory of Neuroscience, IRCCS, Istituto Auxologico Italiano, Milano, Italy; 3 PhD Program in Neuroscience, School of Medicine and Surgery, University of Milano-Bicocca, Milan, Italy; 4 Department of Pathophysiology and Transplantation, “Dino Ferrari Center”, Università degli Studi di Milano, Milano, Italy; 5 Institute of Diagnosis and Health, IDC-Hermitage Capodimonte, Naples, Italy; 6 Department of Medicine, Surgery and Dentistry, University of Salerno, Salerno, Italy; 7 Department of Motor Sciences and Wellness, University “Parthenope”, Naples, Italy

**Correction to: Neurological Sciences (2023)**

10.1007/s10072-023-06619-w

The initial online version has been corrected. More specifically, additional statements have been added to the Funding and Conflict of Interest sections and an Online Supplementary Material has been removed because it has been provided only for peer-review purposes and not for publication.

The original article has been corrected.

