# Supplementary material for: Psychometrics and diagnostics of the Italian version of the Beck Depression Inventory-II (BDI-II) in Parkinson’s disease
Source: Neurol Sci. 2023 Jan 18;44(5):1607–12. doi: 10.1007/s10072-023-06619-w (PMC10102079; doi:10.1007/s10072-023-06619-w)
Supplement: Supplementary file 1 — (DOCX 16 kb) [file 10072_2023_6619_MOESM1_ESM.docx]

|  | Factor 1 | Factor 2 |
| --- | --- | --- |
| Loss of pleasure | **0.758** | -0.086 |
| Concentration difficulties | **0.724** | 0.006 |
| Loss of interest | **0.682** | -0.195 |
| Indecision | **0.653** | 0.124 |
| Loss of energy | **0.611** | -0.038 |
| Pessimism | **0.568** | 0.322 |
| Devaluation | **0.530** | 0.285 |
| Fatigue | **0.484** | 0.187 |
| Self-criticism | **0.464** | 0.189 |
| Failure | **0.442** | -0.246 |
| Self-dislike | **0.401** | 0.063 |
| Loss of sexual interest | **0.371** | 0.061 |
| Feeling of punishment | **0.359** | -0.253 |
| Agitation | 0.278 | **0.469** |
| Irritability | 0.247 | **0.525** |
| Sadness | 0.177 | **0.567** |
| Feeling of guilt | 0.154 | **0.298** |
| Changes in sleeping pattern | 0.103 | **0.379** |
| Suicidal thoughts | 0.064 | **0.358** |
| Changes in appetite | 0.017 | **0.444** |
| Crying | -0.271 | **0.660** |
| Variance explained (%) | 40.04 | 18.11 |
| Cronbach’s α | 0.830 | 0.700 |

Supplementary Material 2. Regularized Exploratory Factorial Analysis for the BDI-II.

Primary loadings for each item are displayed in bold.

**p* < .001
